# Liquid crystalline poly(propylene imine) dendrimer-based iron oxide nanoparticles†

**DOI:** 10.1039/c9ra03732b

**Published:** 2019-07-22

**Authors:** M. S. Gruzdev, U. V. Chervonova, V. E. Vorobeva, A. A. Ksenofontov, A. M. Kolker

**Affiliations:** G. A. Krestov Institute of Solution Chemistry of the Russian Academy of Sciences Ivanovo 153045 Russian Federation gms@isc-ras.ru; Zavoisky Physical-Technical Institute, FRC Kazan Scientific Center of Russian Academy of Science Kazan 420029 Russian Federation; Kazan National Research Technological University Kazan 420015 Russian Federation

## Abstract

Poly(propylene imine) dendromesogens (generations from 1 to 4) have been utilized for the synthesis and stabilization of ferrimagnetic Fe_2_O_3_ nanoparticles. Reduction of Fe(iii) with further oxidation of Fe(ii) results in the formation of highly soluble nanocomposites of iron oxides in a dendrimer, which are stable under a wide range of temperatures. The magnetic iron oxide nanoparticles were investigated by MALDI-ToF MS spectrometry and elemental analysis. To establish the type of mesophase, X-ray measurements were performed at different temperatures. The calculations of X-ray results demonstrate a hexagonal columnar packing of the molecules in the mesophase. Observation of the samples by TEM gives information about the size of the compounds as well as direct evidence of the implementation of Fe_2_O_3_ nanoparticles into dendrimers. Physical parameters of the magnetic nanoparticles (magnetic moment, effective magnetic anisotropy) have been determined from analyses of the EPR data.

## Abstract 2

Poly(propylene imine) dendromesogens (generations from 1 to 4) have been utilized for the synthesis and stabilization of ferrimagnetic Fe_2_O_3_ nanoparticles.

## Graphical abstract

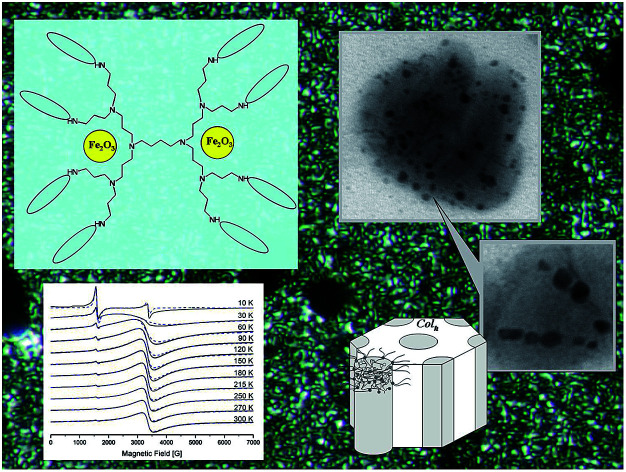

## Introduction

Magnetic materials are key components in modern technology, with applications ranging from data storage^1^ to magnetic resonance imaging contrast agents.^2^ Magnetic iron oxide nanoparticles (MIO NPs) of different appropriate surface modifications can be prepared using diverse physical and chemical methods.^3^ Colloidal iron oxide particles (magnetite and maghemite) are generally known for their use in information technology and storage media.^4–8^ However, various forms of iron oxide particles (also called ferrofluids, FFs) are also used for a wide range of chemical and biomedical applications including chemical and bioseparation, diagnostics, magnetic resonance imaging (MRI), magnetic fluid hyperthermia, and targeting and localization of cytotoxic and radiotherapeutic drugs.^9–11^ Magnetic particles currently employed in biomedical applications are based on ferromagnetic and superparamagnetic nanoparticles of iron oxide (magnetite Fe_3_O_4_, or maghemite γ-Fe_2_O_3_, mostly 2–30 nm). The particles are usually stabilized by either their surface charge or with suitable stabilizers. Stabilization usually means coating the particles with, or encapsulation in, various surfactants or polymers, *i.e.* forming inorganic–organic nanocomposites, or magnetic nano- and microcapsules.

Recent work with dendrimers has suggested that they can act effectively as organic matrices for the synthesis of inorganic nanoparticles.^12–14^ Dendrimers have attracted the attention of many researchers due to their unique and tunable 3D architectures and highly branched macromolecular characteristics.^15,16^ These physicochemical properties enable dendrimers to be used as a multifunctional nanoplatform for the synthesis of various dendrimer-based MIO NPs for different biomedical applications.

According to the literature,^17^ dendrimer-based MIO NPs can be synthesized using different strategies: (1) dendrimer-stabilized MIO NPs (DSNPs), whereby dendrimers are used as stabilizers to surround or protect the MIO NPs, and the MIO NPs are formed *in situ*;^18,19^ (2) dendrimer-assembled MIO NPs (DANPs), where functionalized dendrimers are assembled onto the surface of preformed MIO NPs *via* specific physical or chemical interactions;^20–22^ (3) dendrimer-entrapped MIO NPs (DENPs), in which the dendrimers are used as templates to synthesize MIO NPs *in situ*;^23–26^ (4) MIO NP-cored dendrimers, whereby the dendrimers grow from the MIO NP cores *via* a step-wise divergent synthesis approach;^27^ and (5) final position dendron-assembled MIO NPs, where dendrons are assembled or grafted onto the preformed MIO NPs.^28,29^

Recently a unique interior structure of dendrimers was suggested allowing them to be used as templates to entrap different metal and semiconductor NPs. In our previous works, to create dendritic nanocomposites or DENPs, Domracheva *et al.*^24–26^ developed a convenient approach according to which, at first complexes of Fe(iii) ions with liquid-crystalline second generation poly(propylene imine) (PPI) dendrimers reduce by N_2_H_2_ Fe(iii) to Fe(ii) then react with NaOH to obtain dendrimer-Fe(OH)_2_ intermediates, that finally oxidize to form the target dendrimer-entrapped Fe_2_O_3_ NPs. In works of Domracheva and co-authors^30,31^ the observation of quantum size effects was described: influence of the size of the semiconductor γ-Fe_2_O_3_ NPs on optical properties and influence of pulsed laser irradiation on superparamagnetic properties of γ-Fe_2_O_3_ NPs. It was recently described that five dendritic complexes from the first to the fifth generations have been synthesized by complex formation between iron(ii) salt and organic dendrimeric ligand.^32^ The given complexes have been shown to form a Col_h_ mesophase with transition to the glass state on cooling.

Motivation for this work arises from our desire to find a suitable method to fabricate dendrimer-entrapped Fe_2_O_3_ NPs from iron(iii)-containing dendritic complexes. Our aim was to synthesize stable nanoparticles Fe_2_O_3_ into poly(propylene imine) dendrimer as well as to investigate the structure and the mesomorphic properties of this compound. We describe, herein, the synthesis, characterization, liquid-crystalline properties, and supramolecular organization of the magnetic iron oxide nanoparticles in a liquid-crystalline poly(propylene imine) dendrimer and physical parameters of the magnetic nanoparticles (magnetic moment, effective magnetic anisotropy).

## Experimental

### Materials and methods

All solvents which were used for the synthesis such as benzene, ethanol, tetrahydrofuran (THF), methylene chloride (DCM) and chloroform are available from Merck. Teflon filters on a syringe (200 nm and 450 nm mesh) are from ROTH Rotilabo PTFE.

Gel-permeation chromatography was performed by means of the following chromatography setup: SDV-columns (30 × 80 mm, 5 nm particle size) 102, 103, 104 Å pore size from Polymer Standards Service (PSS). Pump: Spectra Physics P100; UV-detector: Waters 440, *λ* = 254 nm; IR-detector: Waters 410. Flow-rate: 0.5 ml min^−1^. Eluent: THF with 0.25 wt% tetrabutylamonium bromide; internal standard 1,2-dichlorobenzene.

Mass-spectra were obtained by MALDI-ToF method (Matrix Assisted Laser Desorption/Ionization Time-of-Flight mass spectrometry) on a spectrometer Brucker Reflex™ III; matrix: 7-hydroxycoumarin.

UV/Vis-spectra were registered in solutions of methylene chloride and tetrahydrofuran (0.5 mg/10 ml) by a spectrophotometer Hitachi U-3000 using 1 cm quartz cells. Fluorescence spectra were recorded on a Shimadzu RF-5301 fluorometer, using a quartz cell of 1 cm path length. The concentration of the solution was 0.5 mg of substance in 10 ml of solvent. The velocity of the spectrum recorder was 0.1 nm s^−1^. The coefficient of limiting diaphragm was 0.5.

FT-IR spectra of the compounds were recorded on a BioRad Digilab FTS-40 device in the region of 4000–350 cm^−1^ on SiO_2_ disk.

Differential scanning calorimetry measurements were carried out on two machines: PerkinElmer Diamond DSC and NETZCH DSC 204 F1 device in aluminium capsules; the weight of the sample ≈10 mg, the heating rate was 10 °C min^−1^ in N_2_ atmosphere.

The phase transition behaviour of Fe(ii) complexes was observed by means of a polarizing microscope Nikon Diaphot 300 equipped with a hot stage Mettler FP 90.

Thermogravimetric analysis was performed on the device TGA/SDTA851 from Mettler Toledo, the heating rate was 10 °C min^−1^ in N_2_ atmosphere.

X-ray diffraction measurements were carried out with a Guinier goniometer Huber 600, a monochromator Huber 611 with a generator Seifert (CuK_α1_, *λ* = 1.54051 Å) and a temperature controller Huber HTC 9634.

The EPR experiments were carried out on the powder samples using the X-band (9.41 GHz) CW-EPR EMXplus Bruker spectrometer equipped with the helium ER 4112 HV and the digital ER 4131VT temperature control systems.

Elemental analysis was carried out for the following elements: C, H, N, Cl, Fe in two special laboratories – Mikroanalytisches Labor I. Beetz (Kronach) and Mikroanalytisches Labor E. Pascher (Remagen, Germany).

The transmission electron microscopy (TEM) experiment was performed using a “TEM ZEISS902” electron microscope operating at 80 kV. The sample was prepared by coating a film of a substance from the solution to the surface of a copper-carbon plate. Micrographs of solutions of the studied substances were prepared on a “LEO OMEGA 922” electron microscope (200 kV) by freezing the dissolved sample.

### Synthetic procedure

Liquid crystal poly(propylene imine) dendrimers with “two-chain” groups of 3,4-bis(decyloxy)benzoate from the first to the fourth generations were synthesized by method which has already been reported.^33^ Dendrimeric iron(iii) complexes from the first to the fourth generation of 3,4-bis-(decyloxybenzoyl) poly(propylene imine) derivatives were synthesized according to our previously publications.^24,32^ The general procedure of preparing dendrimer-entrapped Fe_2_O_3_ NPs is described for dendrimer D1-K2.10-(Fe_2_O_3_)_1_. The amount of the starting compounds and precursors is given in Table SI 1 of ESI.†

#### Synthesis of D1-K2.10-(Fe_2_O_3_)_1_ (1)

**Scheme 1:**
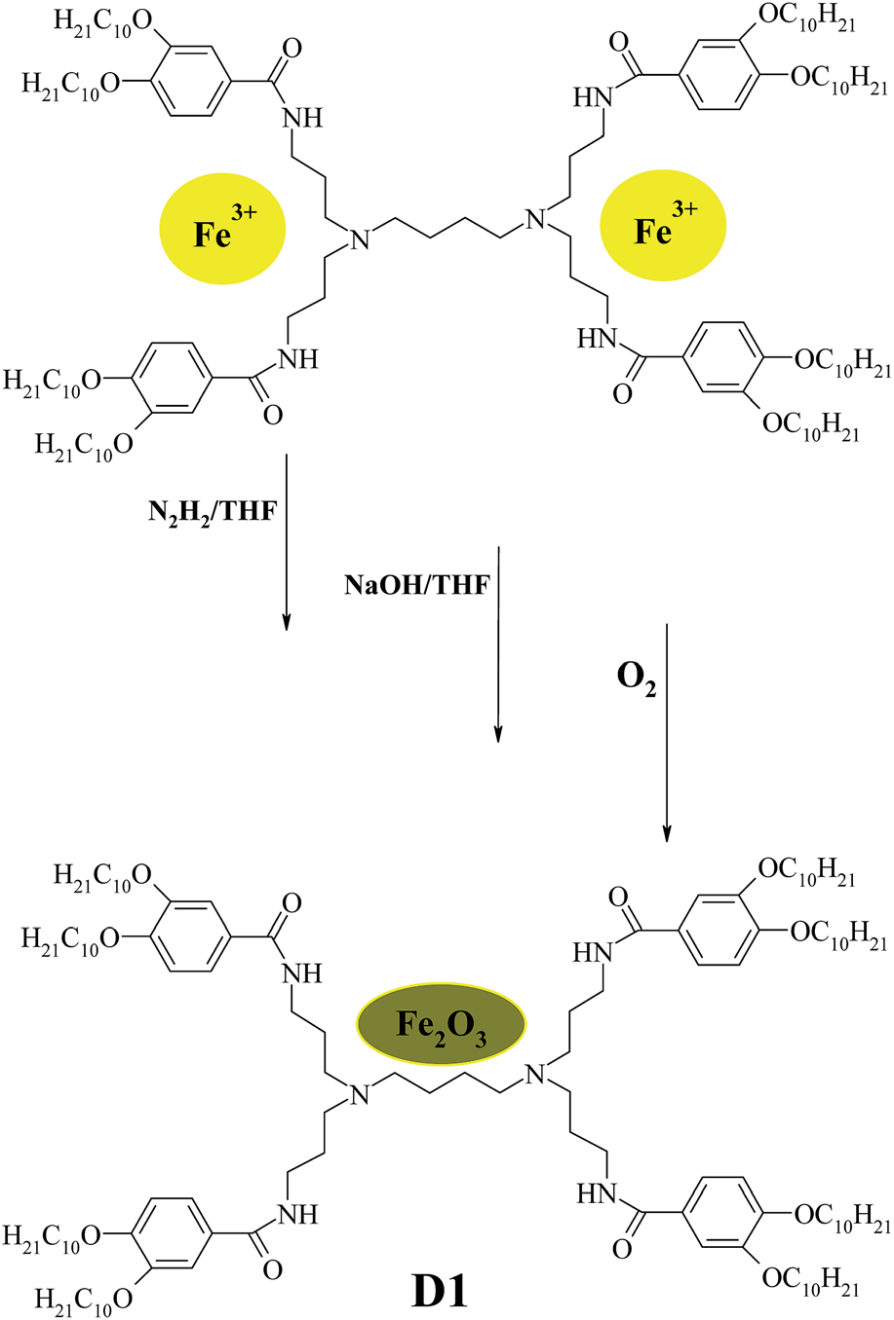
Synthesis of 3,4-bis-(decyloxybenzoyl) poly(propylene imine) dendrimer Fe_2_O_3_ NPs of the first generation D1-K2.10-(Fe_2_O_3_)_1_ (D_1_).

It was synthesized according to Scheme 1. In a flask with a mechanical stirrer, 50 ml of solution of the first generation dendrimeric iron(iii) complex (200 mg; 0.1 mmol) in THF was quickly degassed and put under Ar. Afterwards, degassed 1 M N_2_H_4_ (96 ml; 30 mmol) in THF was added and the mixture was stirred for 1.5 hours. The color of the solution changed from brown to yellowish-green, which indicates the reduction of Fe^3+^ to Fe^2+^. Addition of NaOH (60 mg; 15 mmol) in THF to the above solution resulted in the change of color from yellowish-green to olive-green, indicating the formation of Fe(OH)_2_. The mixture was stirred under Ar for 2 hours and bubbled by O_2_ for 2.5 hours. The color instantly changed to reddish-brown (immediately). The mixture was filtered off on a glass filter with porosity 4. The solid residue on the filter was washed with cold ethanol until neutral pH, dissolved in 100 ml THF and filtered through a 450 mesh PTFE filter. The clear solution was evaporated to dryness. The solid residue was dissolved in 50 ml benzene, filtered through a 200 nm mesh PTFE filter and lyophilized by evaporating benzene from the still cold, solid sample under a fine vacuum (2 × 10^−2^ mbar). Yield: 78.7%. Elemental analysis (%): calculated for C_124_H_216_N_6_O_15_Fe_2_: C 69.51; H 10.16; N 3.92; O 11.2; Fe 5.21. Found: C 69.99; H 10.45; N 4.04; O 10.39; Fe 5.13.

#### Synthesis of D2-K2.10-(Fe_2_O_3_)_2_ (2)

Methodology is similarly to D1-K2.10-(Fe_2_O_3_)_1_. Yield: 58.3%. Elemental analysis (%): calculated for C_256_H_448_N_14_O_30_Fe_4_: C 69.47; H 10.2; N 4.43; O 10.85; Fe 5.05. Found: C 69.99; H 10.45; N 4.04; O 11.36; Fe 5.11.

#### Synthesis of D3-K2.10-(Fe_2_O_3_)_5_ (3)

Methodology is similarly to D1-K2.10-(Fe_2_O_3_)_1_. Yield: 47.9%. Elemental analysis (%): calculated for C_520_H_912_N_30_O_63_Fe_10_: C 68.28; H 10.04; N 4.59; O 11.01; Fe 6.01. Found: C 69.01; H 10.07; N 4.43; O 10.36; Fe 6.13.

#### Synthesis of D4-K2.10-(Fe_2_O_3_)_10_ (4)

Methodology is similarly to D1-K2.10-(Fe_2_O_3_)_1_. Yield: 38.7%. Elemental analysis (%): calculated for C_1048_H_1840_N_62_O_126_Fe_20_: C 68.25; H 10.06; N 4.71; O 10.93; Fe 6.06. Found: C 68.41; H 10.04; N 4.68; O 10.86; Fe 6.01.

## Results and discussion

### General procedure of synthesis of dendrimer-entrapped MIO NPs (DENPs)

**Scheme 2:**
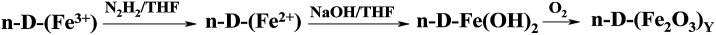
Schematic presentation of procedure for synthesized DENPs.

The preparation of dendrimer iron oxide nanoparticles from the first to the fourth generation (D1–D4) are depicted in Scheme 2.

The synthesis was carried out in three steps in a solution of dry THF in an atmosphere of argon. The procedure yields iron hydroxide inside a dendrimer matrix as an intermediate product followed by its conversion to iron oxide by means of oxidation in an oxygen current. Bubbling by oxygen took place at room temperature and atmospheric pressure. When the final stage was completed the reaction mixture was passed through a glass filter with porosity 4 and washed with a small amount of dry tetrahydrofuran.

**Fig. 1:**
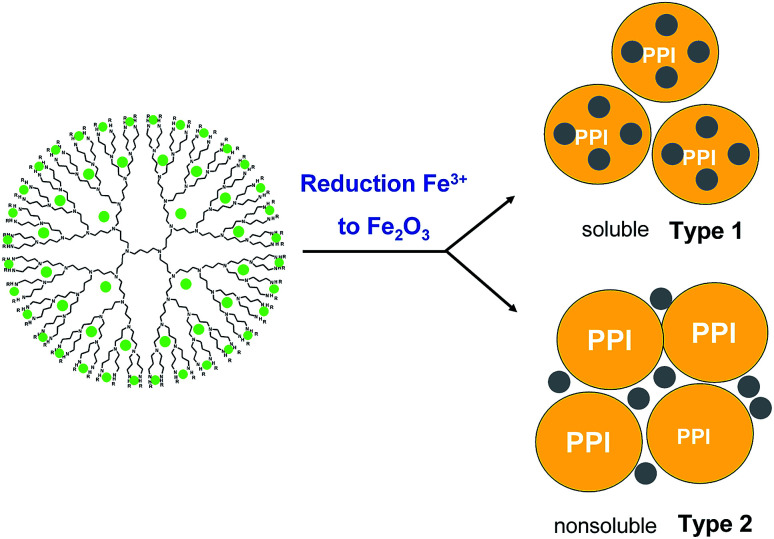
Schematic representation of dendrimer-entrapped MIO NPs with two types of product: soluble (Type 1) and nonsoluble (Type 2) in organic solvents.

In some cases two products of DENPs were obtained: one of them is a pale-yellow solid soluble in tetrahydrofuran (Type 1), and the other is an insoluble (Type 2) yellow-brown precipitate on a glass filter (insoluble product), Fig. 1. The solution was filtered through a 400 nm mesh PTFE filter, concentrated on a rotary evaporator, dissolved in benzene, and filtered through a 200 nm mesh PTFE filter.

The target compounds were isolated by lyophilization from benzene. The first type of products corresponded to nanoparticles of iron oxide inside the dendrimeric structure with an oxide particle size of no more than 4 nm. It is soluble in tetrahydrofuran, benzene, chloroform, and methylene chloride. Substances are amorphous solid, with colour varying from pale-yellow to dark brown depending on the generation. The insoluble product (Type 2) is a dendrimer surrounded by large (20–30 nm) particles of iron oxide. Its structure will be mentioned below. The completion of the oxidation reaction was evidenced by the changing of the UV spectra, which clearly demonstrates the absence of iron ions in ultimate products.

**Fig. 2:**
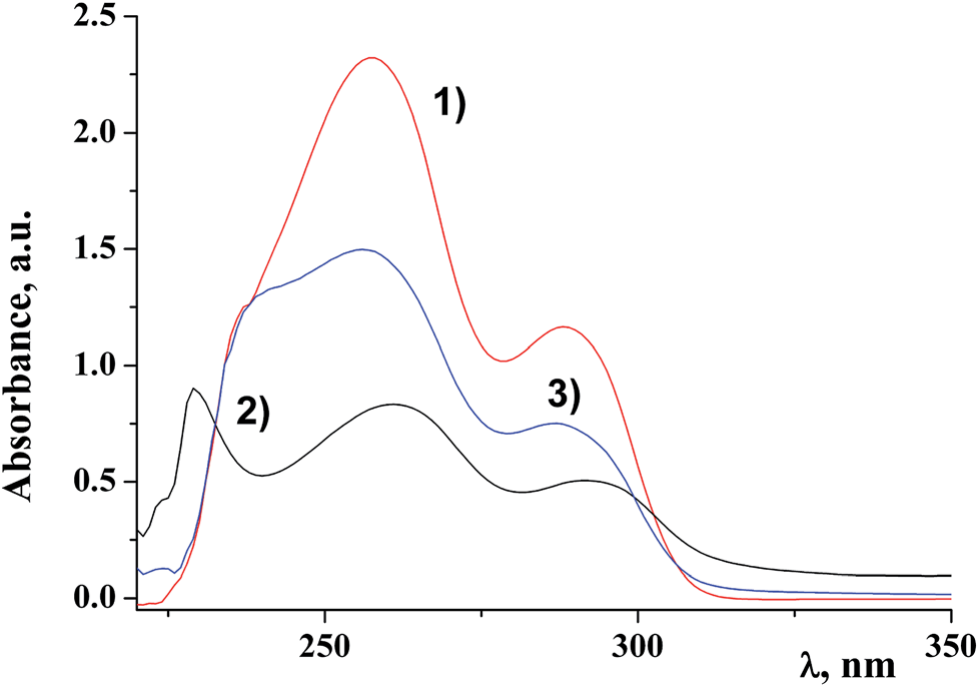
UV-spectra of the ligand, complex and DENPs of the first generation. The concentration is 0.05 g l^−1^ in CH_2_Cl_2_. (1) Free metal dendrimer; (2) dendritic complex and (3) nanocomposite D1-K2.10-(Fe_2_O_3_)_1_.

Fig. 2 shows electronic absorption spectra of the ligand, the dendritic complex and the dendritic nanocomposite of the first generation D1-K2.10-(Fe_2_O_3_)_1_.

It is clearly seen that the nanocomposite is an individual compound and the final reaction is fully completed with a qualitative yield. The data of the electronic absorption spectra of DENPs are given in the Table 1. Analysis of the data suggests that π–π* transitions of aromatic rings of the dendritic periphery hardly undergo any changes (*λ*_1_ and *λ*_2_). At the same time the value *λ*_3_ shifts to the UV region.^34^ It can be concluded that iron oxide nanoparticles are situated on the border of the dendritic periphery and the core.

**Table 1:** Absorption spectra of DENPs in methylene chloride

Compound	*λ*_1_, nm	*λ*_2_, nm	*λ*_3_, nm
D1-K2.10-(Fe_2_O_3_)_1_	239.4	255.8	285.9
D2-K2.10-(Fe_2_O_3_)_2_	—	254.2	286.9
D3-K2.10-(Fe_2_O_3_)_5_	230.5	250.8	287.8
D4-K2.10-(Fe_2_O_3_)_10_	230.5	257.4	288.5

Unfortunately, no use can be made of the NMR spectroscopy for structural determinations because of the paramagnetic nature of magnetic iron oxide nanoparticles.

### Gel permeation chromatography

**Fig. 3:**
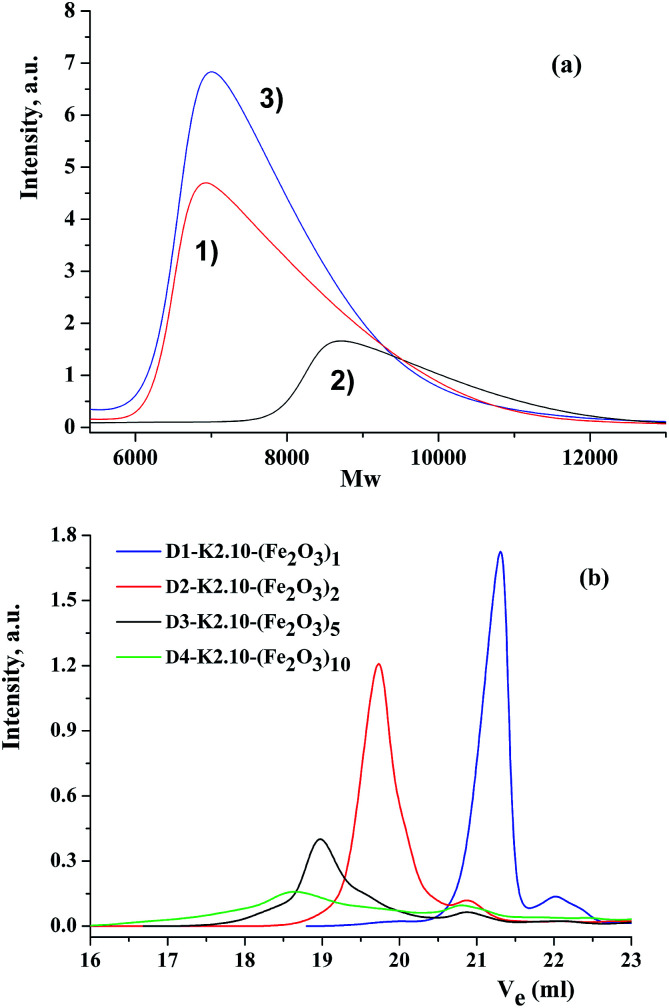
(a) GPC-chromatograms of the complex, nanocompound and ligand; (b) GPC-chromatograms of nanocompounds from the first to the fourth generation. (1) Free metal dendrimer; (2) dendritic complex and (3) nanocomposite D1-K2.10-(Fe_2_O_3_)_1_.

The purity and individuality of the oxide-containing compounds obtained was verified by gel-permeation chromatography. As an example, three chromatograms are presented in Fig. 3a: DENP of the first generation (D1-K2.10-(Fe_2_O_3_)_1_), the complex and the ligand. Comparing these chromatograms one can conclude that all three compounds have different separation and different molecular weights and as a result, various volumes of the eluent used.

It also confirms that the final reaction of obtaining DENPs proceeds completely and with qualitative and quantitative yield. Gel-permeation chromatograms of dendrimers from the first to the fourth generations are given in Fig. 3b. The main parameters of chromatograms of dendritic nanocomposites are combined in Table 2.

**Table 2:** Parameters of GPC-chromatogram of dendritic nanocomposites^a^

Compound	*M*_n_, g mol^−1^	*M*_w_, g mol^−1^	*M*_z_, g mol^−1^	*V*_p_, ml	*M*_p_, g mol^−1^
D1-K2.10-(Fe_2_O_3_)_1_	6.5 × 10^3^	7.37 × 10^3^	8.15 × 10^3^	21.3	7 × 10^3^
D2-K2.10-(Fe_2_O_3_)_2_	2.77 × 10^3^	1.59 × 10^4^	2.01 × 10^4^	19.7	1.97 × 10^4^
D3-K2.10-(Fe_2_O_3_)_5_	4.09 × 10^3^	2.24 × 10^4^	3.39 × 10^4^	18.9	3.21 × 10^4^
D4-K2.10-(Fe_2_O_3_)_10_	2.3 × 10^3^	2.62 × 10^4^	6.24 × 10^4^	17.6	3.95 × 10^4^

a *M*_n_ – average number of molar weight; *M*_w_ – average mass number of molar weight; *M*_z_ – average molar weight; *V*_p_ – volume of the eluent; *M*_p_ – maximum of average molar weight. Eluent: THF with 0.25 wt% tetrabutylamonium bromide; internal standard 1,2-dichlorobenzene.

### FT-IR spectroscopy

**Fig. 4:**
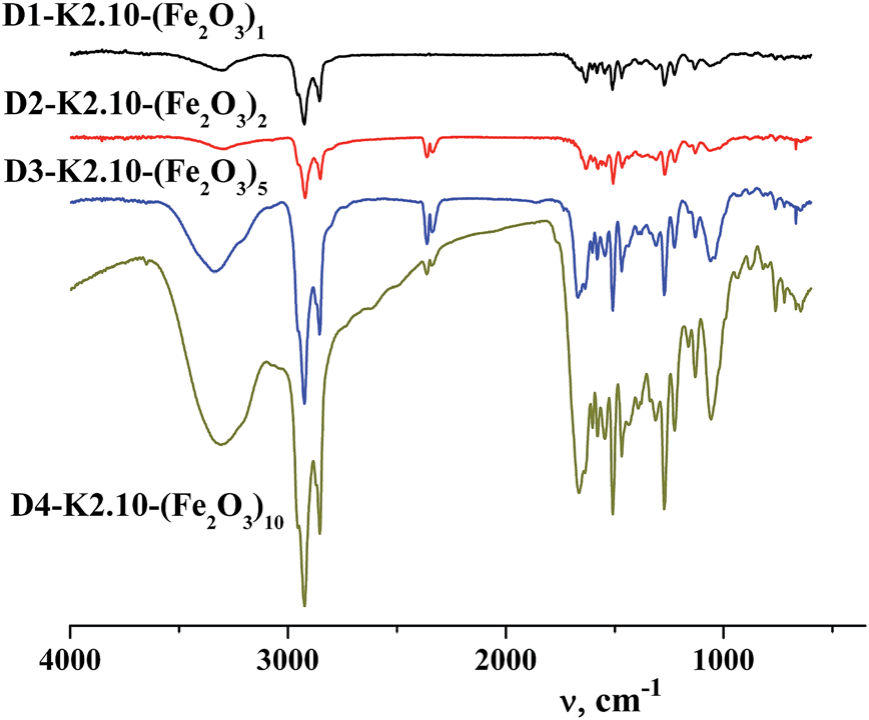
FT-IR spectra of DENPs from the first to the fourth generation.

Analysis of the IR spectra of the dendritic homologous confirmed the proposed structure of the synthesized compounds. In the range of stretching vibrations 2853–2954 cm^−1^ absorption bands of –CH_2_–, –CH_3_ groups of alkyl hydrocarbonic chains on the dendritic periphery are observed, Fig. 4. The absorption band in the region of 722 cm^−1^ is referred to pendular oscillations of CH_2_– groups of long chains. Deformation vibrations of hydrocarbonic fragments appear at 1466 cm^−1^.^35^ Average intensity of the absorption band in the region of 1636 cm^−1^ for homologues of this series of compounds corresponds to the frequency of stretching vibrations of the C=O amide group. In the IR spectra of compounds there are intense absorption bands at 1601, 3072 cm^−1^ which indicate the presence of aromatic rings. The occurrence of absorption bands of deformation out-of-plane vibrations of the C–H groups of the benzene ring was found at 862 cm^−1^ which is characteristic of 1,3,4-substitution of the aromatic ring. In accordance with the chemical structure of the dendritic core (poly(propylene imine)) a few absorption bands of all four types of amides are observed in the FT-IR spectra. Similarly, a narrow, clearly defined medium intensity band corresponding to “Amide I” is situated at 1579 cm^−1^. The high intensity band belonging to “Amide II” is located in the range of 1506 cm^−1^. The bands relating to the characteristic vibrations of “Amide III” lie at 1272, 1311, 1434–1466 cm^−1^. It is to be noted that intensity of the “Amide III” bands changes from high to low and the width of these bands also changes which is characteristic of this type of amide group. The two narrow bands of “Amide IV” are at 721–723 and 762 cm^−1^ and are characterized by low intensity.^36^ In the region of 1137 cm^−1^ a narrow band is observed relating to –N–C– vibrations of propylene imine.

A broad, low intensity band of intermolecular hydrogen bond characteristic of associates is present in the region of 3294 cm^−1^. A distinctive feature of the IR spectra of dendritic nanocomposites is the absence of absorption bands of stretching vibrations of the complex in the range 2491–2611 cm^−1^. In contrast to dendritic complexes DENPs have absorption bands at 2721 and 2797 cm^−1^ indicating their similarity to dendritic ligands, namely the absence of any intramolecular complexes. Thereby absorption bands in FT-IR spectra, Fig. 4, confirm the proposed structure of synthesized dendritic nanocompounds with encapsulated nanoparticles of iron(iii) oxide. From the FT-IR spectroscopy data it can be concluded that no destruction (self hydrolysis, decomposition of periphery) of dendrimers used by us and DENPs obtained on the basis of them occurs during the synthesis.

Changes in the FT-IR spectra of MIO NPs in the range of 1135–929 cm^−1^ accompanied by an increase in the intensity of the new band (1056 cm^−1^) indicate the presence of iron oxide inside the dendritic matrix and a growth in its content in the samples depending on the generation.

### MALDI-ToF mass-spectrometry

The stability of the obtained oxide-containing compounds as well as the presence of iron oxide inside the dendritic matrix were confirmed by mass-spectrometry data (MALDI-ToF MS). Table 3 shows the values of the main molecular ions of nanocomposites.

**Table 3:** Mass spectrometric data of dendritic nanocomposites^a^

Compound	Molecular ions	
	Calculated	Found
D1-K2.10-(Fe_2_O_3_)_1_	1983 [L]*	1984.9 [L + 1]^+^**
	2055 [L·FeO]*	2056.9 [L·FeO + 2]^+^**
	2142 [L·Fe_2_O_3_]*	2146.5 [L·Fe_2_O_3_ + 4]^+^**
D2-K2.10-(Fe_2_O_3_)_2_	4106 [L]*	4105.9 [L-1]^+^**
	4266 [L·Fe_2_O_3_]*	4266.1 [L·Fe_2_O_3_]^+^**
	4448 [L·Na·2(Fe_2_O_3_)]*	4449.1 [L·Na·2(Fe_2_O_3_) + 1]^+^**
D3-K2.10-(Fe_2_O_3_)_5_	8353 [L]*	8354.6 [L + 1]^+^**
	8513 [L·Fe_2_O_3_]*	8517.7 [L·Fe_2_O_3_ + 4]^+^**
D4-K2.10-(Fe_2_O_3_)_10_	16 846 [L]*	16 843.9 [L − 3]^+^**
	17 006 [L·Fe_2_O_3_]*	—

a * – hypothetical composition of the molecular ion of ligand [L] with inclusion of proposed nanoparticles [L·Fe_2_O_3_], ** – composition of the experimental molecular ion [L + *n*]^+^ with nanoparticles [L·Fe_2_O_3_ + *n*]^+^, where *n* – the difference between the theoretical and experimental values.

**Fig. 5:**
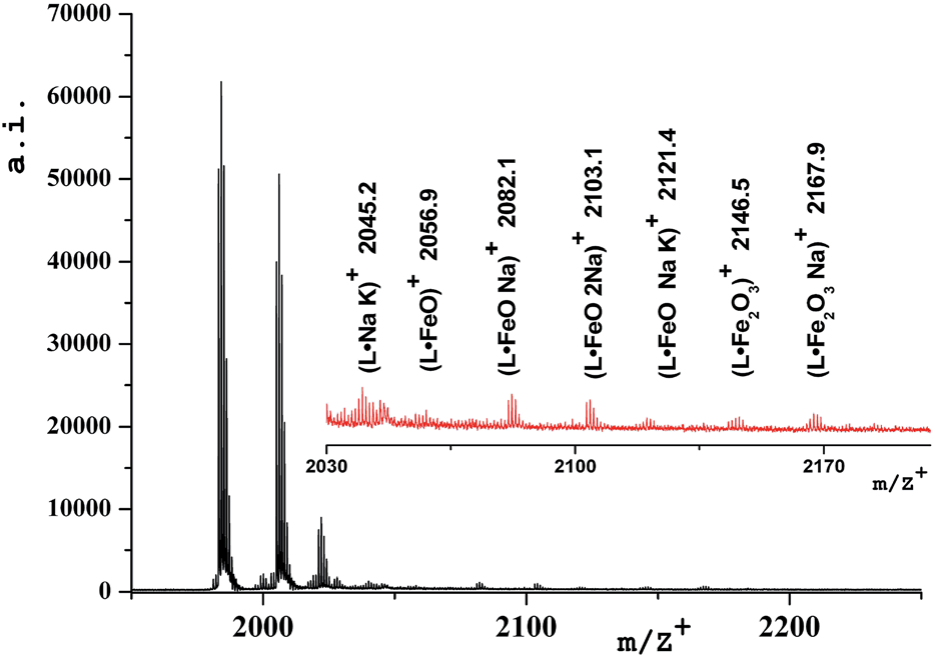
Mass spectrum of compound D1-K2.10-(Fe_2_O_3_)_1_.

Fig. 5 shows the mass-spectrum of the first homolog with detailed interpretation of the present molecular ions. It was established that dispersibility of the compounds increases with the growth of the generation number and this leads to the fact that no molecular ions can be clearly detected for higher generations, starting from the third. In mass spectra of DENP there are molecular ions corresponding to the presence of iron oxide inside the dendritic matrix. In the case of a physical mixture of iron oxide and a dendrimer only the molecular ion of the dendrimer will be present in the mass spectrum of such a product and molecular ions corresponding to the iron oxide give signals in the region of 160–500 units.

We have established that the gamma form of iron oxide (γ-Fe_2_O_3_) can produce large molecular aggregates and turn into another form under laser irradiation.^37^ It is precisely this fact that explains the presence of a molecular ion 2056.9 [M^+^L·FeO] in the mass spectrum of the DENP of the first generation, Table 3.

**Fig. 6:**
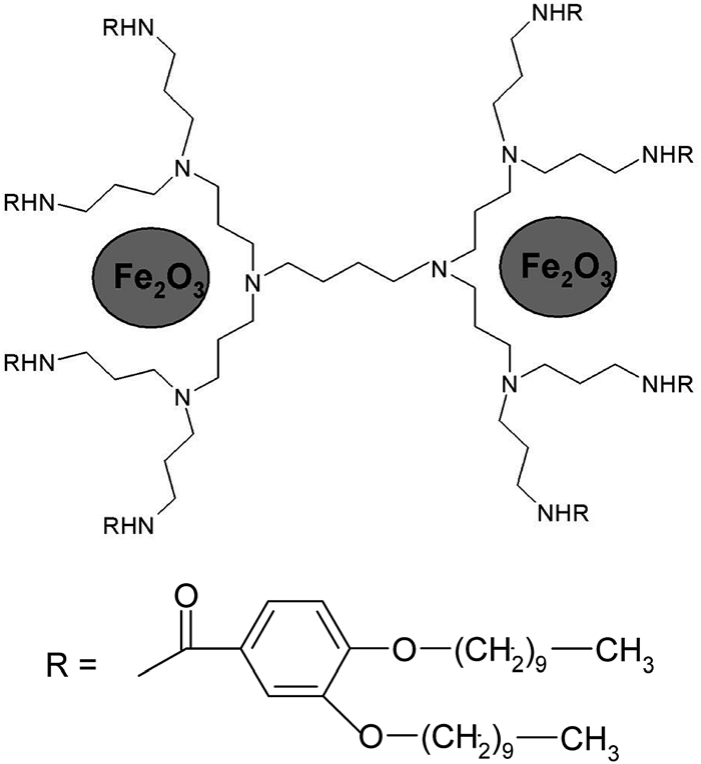
Hypothetical model of compound D2-K2.10-(Fe_2_O_3_)_2_ on the basis of mass spectrometry.

The occurrence of molecular ions of the composition “dendrimer–iron oxide” in soluble dendritic nanocomposites is the last argument to prove the existence of their stable forms, Fig. 6. It becomes obvious that the nanocomposite dendrimer between the branches of which nanoparticles of iron oxide are situated. In agreement with the data of mass spectra and elemental analysis, we can predict the approximate number of iron oxide molecules which accrue on one dendrimer molecule depending on the generation number. Thus, it was found that the first generation macromolecule includes 1 molecule of γ-Fe_2_O_3_, the second generation macromolecule includes 2 molecules of γ-Fe_2_O_3_, the third – 5 molecules, and the fourth – 10 molecules of iron oxide.

**Fig. 7:**
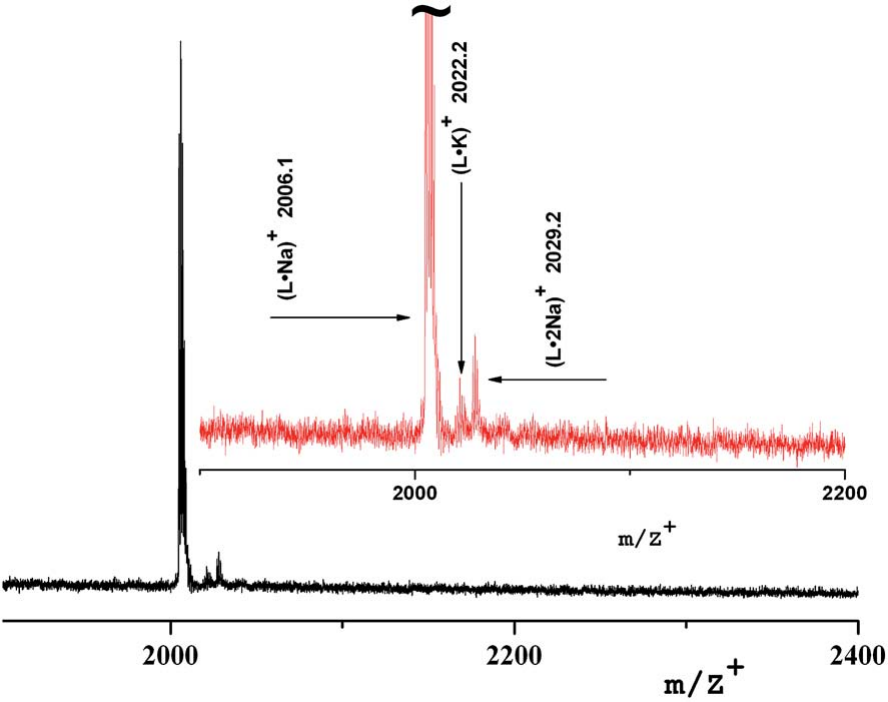
Mass spectrum of insoluble nanocomposite of the first generation (Type 2).

The mass spectrum of the insoluble compound (Type 2) has other characteristics: as a first generation sample there is only a molecular ion 2029.9 [L·Na]^+^ corresponding to a ligand with Na, Fig. 7. It can be assumed that it is a dendrimer surrounded by large particles of brown γ-Fe_2_O_3_ of size 20–30 nm.

### Mesomorphic properties

The mesomorphic properties of the materials were investigated by the differential scanning calorimetry (DSC), a polarizing optical microscope (POM), and the X-ray diffraction. The transition temperatures, temperatures of decomposition and corresponding enthalpy values are given in Table 4. All transition temperatures of the compounds were obtained on heating with a rate of 10 K min^−1^.

**Table 4:** Transition temperatures of dendritic nanocompounds^a^

Compound	*T*_g_, °C	*T*_m_, °C	Δ*H*_m_, kJ mol^−1^	*I*, °C	Δ*H*_iso_, kJ mol^−1^	Phase	*T*_dec_, °C
D1-K2.10-(Fe_2_O_3_)_1_	42.0	87.5	23.5	105.5	53.9	Col_h_	134.5
D2-K2.10-(Fe_2_O_3_)_2_	26.0	45.2	23.1	96.5	66.4	Col_h_	144.0
D3-K2.10-(Fe_2_O_3_)_5_	15.5	47.5	25.5	64.5	89.2	Col_h_	114.5
D4-K2.10-(Fe_2_O_3_)_10_	38.5	61.5	110.4	117.5	125.1	Col_h_	156.5

a *T*_g_ – glass-transition temperature; *T*_m_ – temperature of the mesophase formation; *I* – temperature of the transition from the mesophase to isotrope; *T*_dec_ – decomposition temperature according to the thermogravimetry data; Col_h_ – columnar hexagonal mesophase.

**Fig. 8:**
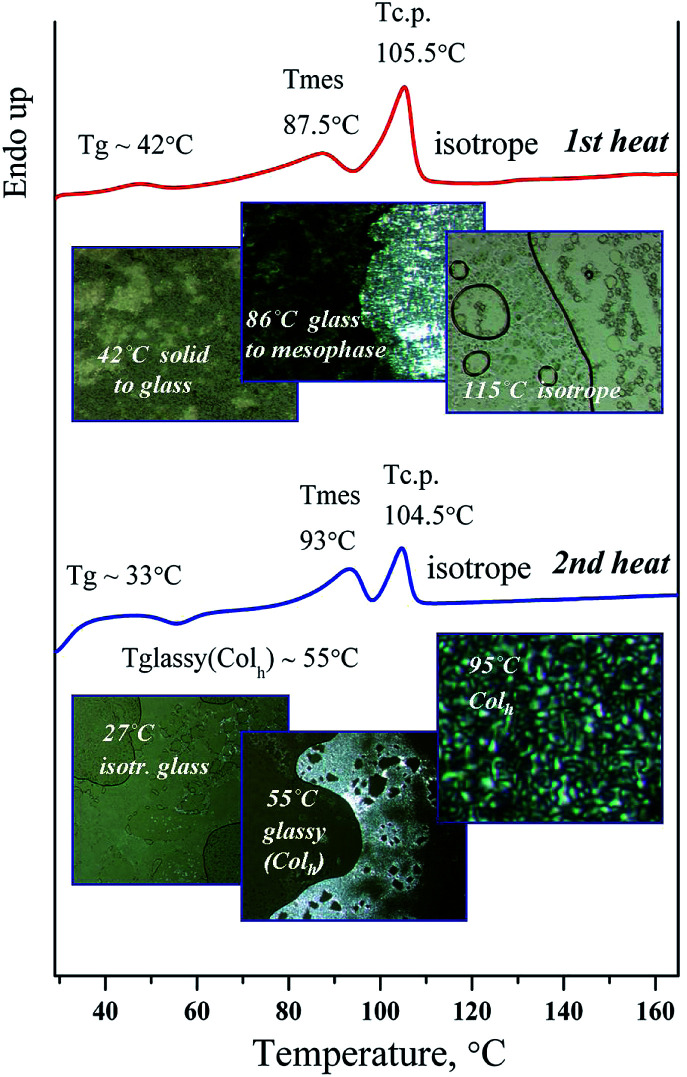
DSC and photographs of D1-K2.10-(Fe_2_O_3_)_1_ in the heating cycle.

As can be seen from Table 4 all of the dendrimers exhibit liquid crystalline behavior. The mesophase textures were observed with a polarizing microscope using thin films of a sample mounted between the glass slide and the cover. The columnar mesophase was easily identified by the optical textures (see Fig. 8). On heating from a solid to the melting point and on cooling from the isotropic phase to the mesophase, they exhibit typical filament domains with a nongeometrical texture and homeotropically aligned areas which appear completely dark, indicating that these mesophases are optically uniaxial as is typical for hexagonal columnar mesophases.

A series of photographs of D1-K2.10-(Fe_2_O_3_)_1_ show the difference between the Col_h_ phase and the crystalline phase, Fig. 8. The complexes demonstrate phase transitions of the “solid to mesophase” and “mesophase to Iso” types with clearing temperatures by POM observation. The phase transition behavior of samples in the 2nd heating process seems to be “glassy(Col_h_) to Col_h_”–“Col_h_ to Isotrope”, with no crystalline phase.

The temperatures of phase transitions of iron oxide-containing dendrimers are shown in Table 4.

The mesomorphic texture in the cooling cycle obtained by polarizing optical microscopy indicates that the mesomorphic ordering remains at room temperature after cooling. Probably it can be explained by forming of the frozen mesophase with ordering appropriate to the Col_h_ mesophase. The texture of the mesophase remains at a reverse transition from the isotropic to mesomorphic state. The transition of “mesophase–solid” is not observed.

Mesophases appeared in the temperature range from 45 to 117 °C in the heating cycle, Table 4. As can be seen from the research results, the thermostability of the mesophase in the synthesized nanocomposites depends on the generation number as well as on the number of mesogenic groups and iron oxide nanoparticles as part of nanocomposite. Using a polarizing microscope it was found that the samples of DENPs under study vitrify at cooling. This is confirmed by the DSC analysis.

In the second scan after the annealing process, very simple thermograms were obtained and only the glass transition could be observed for most of the dendrimers. For this series of dendrimers the *T*_g_ values are reduced from the first to the third generation. Probably this is associated with a disordering effect of the iron oxide nanoparticles in the dendritic matrix. In case of the first three generations the DENPs have a strong effect on the strength of intermolecular interactions of nanocomposites in the mesophase reducing its thermal stability. For dendritic nanocomposite of the fourth generation the contribution to the mesomorphic behavior is determined by a significant increase in the content of mesogenic groups (32) situated on the dendrimer periphery. This leads to a certain enhancement of the *T*_g_ and *T*_m_ values and a considerable expansion of the temperature range of the mesophase existence. The clearing temperature increased with the dendrimer generation. This trend was observed for other liquid-crystalline dendrimers^38^ and confirmed that the stability of mesophases increases with the number of mesogenic units.

To establish the type of the mesophase X-ray measurements were performed at different temperatures, Table 5. The calculations of X-ray results demonstrate the hexagonal columnar packing of the molecules in the mesophases. It is formed invariably depending on the strong interaction between core–core, the column being titled as Col_h_. As described above, DENPs of the first to fourth generations exhibit columnar polymorphism: a *p*2*mg* rectangular columnar mesophase at low temperatures and a *p*6*mm* hexagonal columnar mesophase above the rectangular. The hexagonal and rectangular lattice parameters of the compounds are presented in Table 5.

**Table 5:** Rectangular and hexagonal lattice parameters of DENPs^a^

Compound	*T*/°C	M	Lattice parameter, *a*_h_, *a*_r_, *b*_r_/Å	*N*_h/r_	*S*_h/r_/Å^2^
D1-K2.10-(Fe_2_O_3_)_1_	95	Col_h(o)_	*a*_h_ = 43.6	2.7	1646
	100	Col_r(o)_	*a*_r_ = 122.2	11	601
			*b*_r_ = 54.1		
D2-K2.10-(Fe_2_O_3_)_2_	50	Col_h(o)_	*a*_h_ = 45.5	1.2	1793
	95	Col_r(o)_	*a*_r_ = 133.8	4.3	1535
			*b*_r_ = 49.0		
D3-K2.10-(Fe_2_O_3_)_5_	55	Col_h(o)_	*a*_h_ = 53.7	0.8	2497
	60	Col_r(o)_	*a*_r_ = 133.2	1.9	3039
			*b*_r_ = 52		
D4-K2.10-(Fe_2_O_3_)_10_	65	Col_h(o)_	*a*_h_ = 58.3	0.5	2944
	100	Col_r(o)_	*a*_r_ = 101.2	1.2	5897
			*b*_r_ = 69.1		

a *T* – measurement temperature; *M* – mesophase; *N*_h/r_ – calculated number of molecules per hexagonal or rectangular unit cell; *S*_h/r_ – cross section per column in the hexagonal or rectangular lattice.

The calculated hexagonal parameters *a*_h_ increase linearly with the generation. The volume expansion of the hexagonal lattice with increasing generation is much bigger in the dendrimeric iron oxide nanoparticles than in the ligand, showing the expansion of the dendrimeric framework, though retaining its cylindrical shape and the hexagonal arrangement.

### Supramolecular organization of compounds

**Fig. 9:**
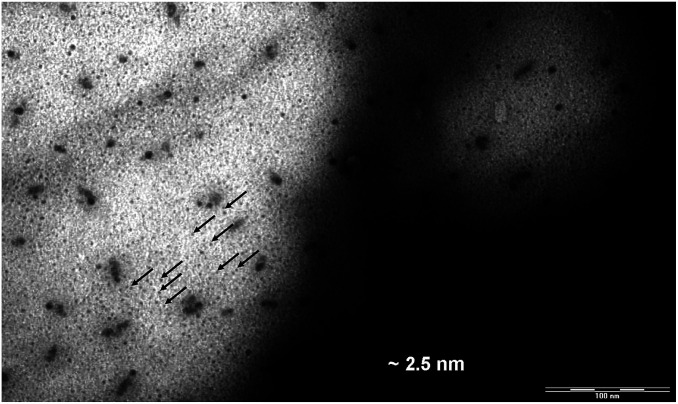
TEM image of D1-K2.10-(Fe_2_O_3_)_1_.

The size of DENPs containing γ-Fe_2_O_3_ was estimated by examining the transmission electron microscopy (TEM). Since the differences in the electron density between the atoms of different parts of the dendrimer (the polyamine core, the benzyl groups, the alkyl shell of the PPI derivatives) are minor, no contrast in the image of a pure dendrimer is observed by TEM. After obtaining the nanocomposite, dark spots appeared in the TEM image. Due to a high electron density of magnetic NPs, their location relative to the selected parts of the dendrimer can be determined. The average diameter, *D*, of iron oxide DENPs (Fig. 9) estimated from the TEM image is about 2.5 nm. The hexagonal lattice constant, *a*_h_ (intercolumnar distance), of the first generation dendrimeric γ-Fe_2_O_3_ composite was calculated from X-ray diffractometry measurements to be about 4.3–4.5 nm, Table 5.

According to our results^24^ and those published by other groups^25,39,40^ it is possible to conclude that maghemite NPs are electrostatically coupled with reactive (amido and amino) nitrogen periphery atoms and incorporated among the branches of a PPI dendrimer.

An electronic photograph of the compound D1-K2.10-(Fe_2_O_3_)_1_ with data on the size of the nanocomposite are given below. For the first generation the size is about 2.5 nm, Fig. 9.

Based on the data obtained, the average sizes of nanocomposites were established to be about 2.5 nm (D1-K2.10-(Fe_2_O_3_)_1_), 3.7 nm (D2-K2.10-(Fe_2_O_3_)_2_), 4.9 nm (D3-K2.10-(Fe_2_O_3_)_5_), 6.1 nm (D4-K2.10-(Fe_2_O_3_)_10_).

**Fig. 10:**
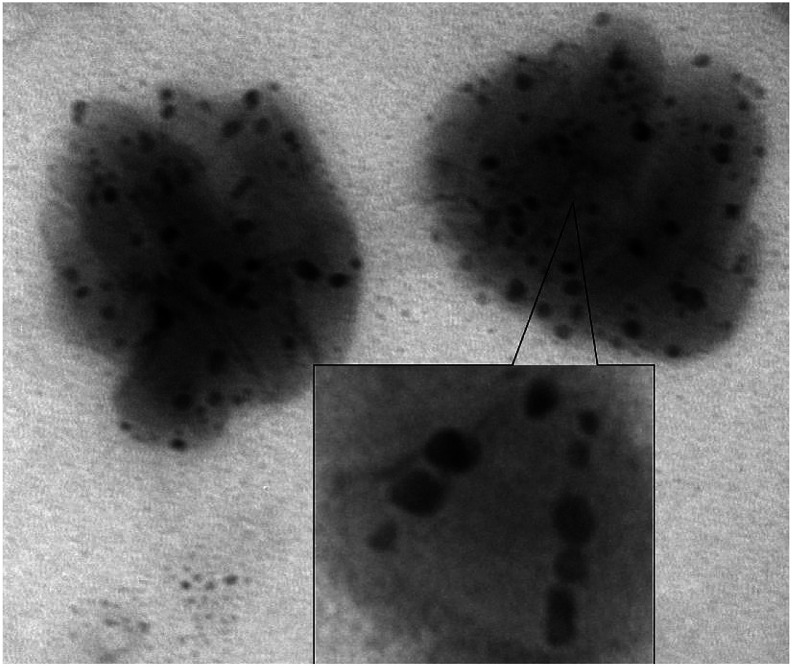
TEM image of D3-K2.10-(Fe_2_O_3_)_5_.

Fig. 10 shows a photograph of assembling nanocomposites with iron oxide nanoparticles D3-K2.10-(Fe_2_O_3_)_5_ and their stacking.

**Fig. 11:**
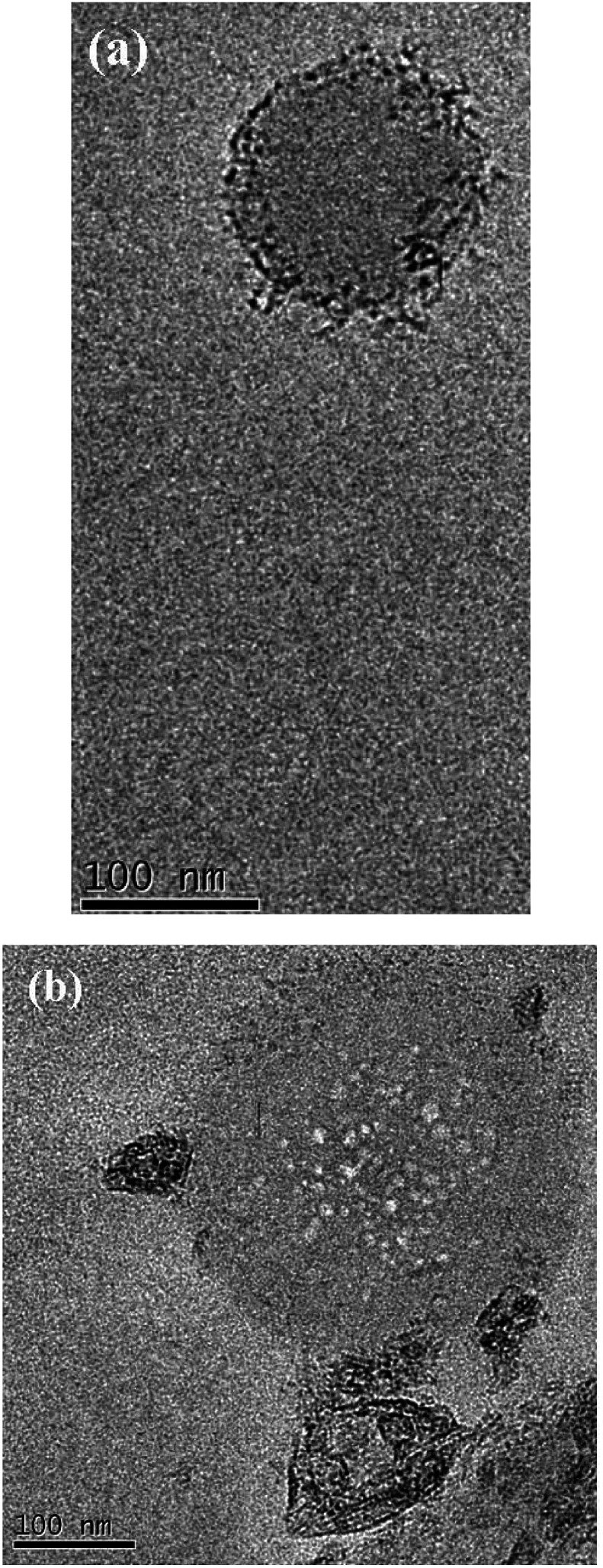
TEM image of the solution of D2-K2.10-(Fe_2_O_3_)_2_ in tetrahydrofuran: (a) aggregate formation, (b) formation of the globules by dendritic molecules.

In the course of further research of dendritic nanocomposites by electron microscopy in instantly frozen solutions it was found that DENPs form high-molecular aggregates. Fig. 11a and b show an example of such a supramolecular assembly.

According to the electron microscopy data and infrared spectroscopy data it can be concluded that aggregation of DENPs proceeds in two ways:

(1) Due to the chemical component – the formation of hydrogen bonds between the associates;

(2) Due to the physical component – the interaction of iron oxide nanoparticles between themselves.

**Fig. 12:**
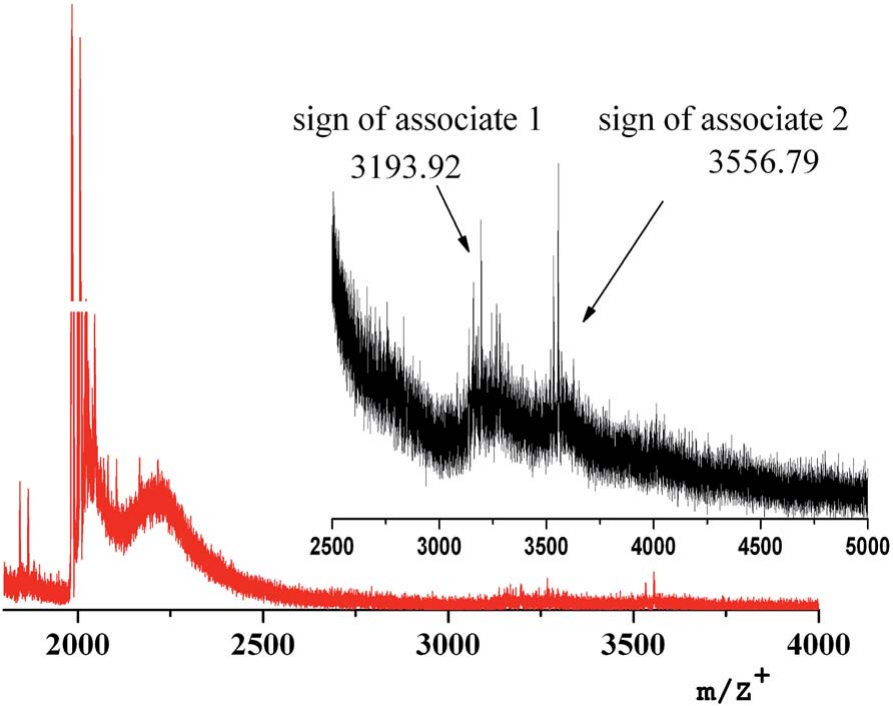
Mass spectrum of D1-K2.10-(Fe_2_O_3_)_1_.

The onset of forming supramolecular aggregates can be registered by the laser mass spectrometry method. For example, two peaks of high molecular ions (3193.92 and 3556.79 *m*/*z*) are in the mass spectrum of the first generation nanocomposite, Fig. 12. Their presence can be explained only by the beginning of the supramolecular aggregation of dendritic nanocomposites in a high-molecular assembly.

### EPR spectroscopy

The electron paramagnetic resonance (EPR) (X-band, *hν* = 0.3 cm^−1^) spectra of polycrystalline samples of generations from the first to the fourth were recorded in a wide range of temperatures from 300 to 4 K. Detailed studies for the second generation were introduced earlier.^25^ All graphs for the first and the third generations are presented in ESI.†

Spectra for the first generation are similar to those of the second, and spectra of the third generation are similar to those of the fourth. Namely, for the first two generations EPR spectra contain only one main signal with effective g-factor *g*_1_ = 2 (I-type), while for the last two – they contain two main signals with effective g-factors: a more intense one with *g*_1_ = 2 (I-type) and a weaker one, with *g*_2_ = 4.3 (II-type). On cooling down, the broad line (I-type) shows a monotonous increase of the linewidth Δ*H*_p–p_ and a shift in the low field. Such behavior is typical for the superparamagnetic resonance of single-domain particles in the absence of transitions to a magnetic ordered state – spin-glass, “stable state”.^41,42^ II-type of iron centres does not shift and does not widen significantly as the temperature decreases. However, for the third and the fourth generations, below approximately 30 K a new resonance appears in the spectra of γ-Fe_2_O_3_, the effective *g* value of which does not depend on temperature and equals 1.99.

Such type of EPR spectrum is known to belong to Fe(iii) ions with the ground state ^6^S_5/2_ (3d^5^) and to be described by the spin-Hamiltonian:^43,44^1
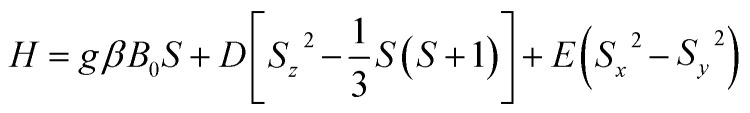
with *g* = 2, *S* = 5/2. The *D* and *E* are the terms of the zero-field splitting, characterizing, respectively, the axial and the rhombic part of distortion of the crystal field from the octahedral (or tetrahedral) symmetry; the relation 0 < *E*/*D* ≤ 1/3 holding.

**Fig. 13:**
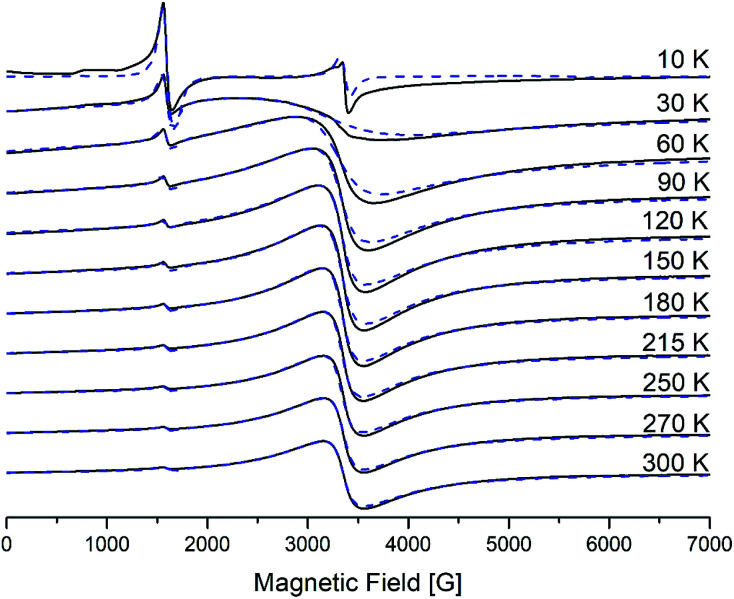
Temperature dependence of EPR spectra for γ-Fe_2_O_3_ NPs encapsulated into the fourth generation dendrimer. All spectra were recorded under the same conditions. Dashed lines are the results of simulation using EasySpin Matlab.

The analysis of the EPR results shows that the observed lines belong to different types of paramagnetic centers. At room temperature the spectra exhibit a have the broad intense signal (I-type) with *g*_1_ = 2 that belongs to Fe(iii) ions in an octahedral environment with a weak (*D* ≪ *gβB*_0_) distorted crystal field, whereas the weaker signal (II-type) with *g*_2_ = 4.3 belongs to iron ions in a tetrahedral environment with a strong (orthorhombic) distorted (*D* ≫ *gβB*_0_, *E*/*D* = 1/3) crystal field. Probably, the octahedral (high-symmetry) centers are located at the border of the dendrimeric core, whereas the tetrahedral centers with a strong rhombic distortion are distributed throughout the branching of the dendrimeric core, Fig. 13, 1 SI and 3 SI.†^24^

The behavior of the third line (*g*_eff_ = 1.99) which appears at low temperatures below 30 K like a typical paramagnetic resonance signal, namely, its amplitude grows and the linewidth decreases as the temperature is reduced. We can assume that this signal may be due to the octahedral symmetry sites of Fe(iii) in the spinel structure of the g-phase. The presence of oxygen vacancies near such centers could break down the exchange interactions that provide the ferrimagnetic properties of the maghemite.^41^ The number of this center is small and we will not consider them in further analysis. The magnetic parameters for II-type of centers do not change with decreasing the temperature.

EPR spectra were simulated by using the EasySpin Matlab numerical program. The results of the simulation are shown by the dashed line on Fig. 13 and are calculated with the following magnetic parameters: *g* = 2.0, *D* = 0.02–0.04 cm^−1^, *E* = 0 cm^−1^ for I-type centers and *g* = 1.97, *D* = 0.42 cm^−1^, *E* = 0.13 cm^−1^ for II-type centers. As can be seen, a satisfactory agreement is obtained between the experimental and theoretical spectra.

**Fig. 14:**
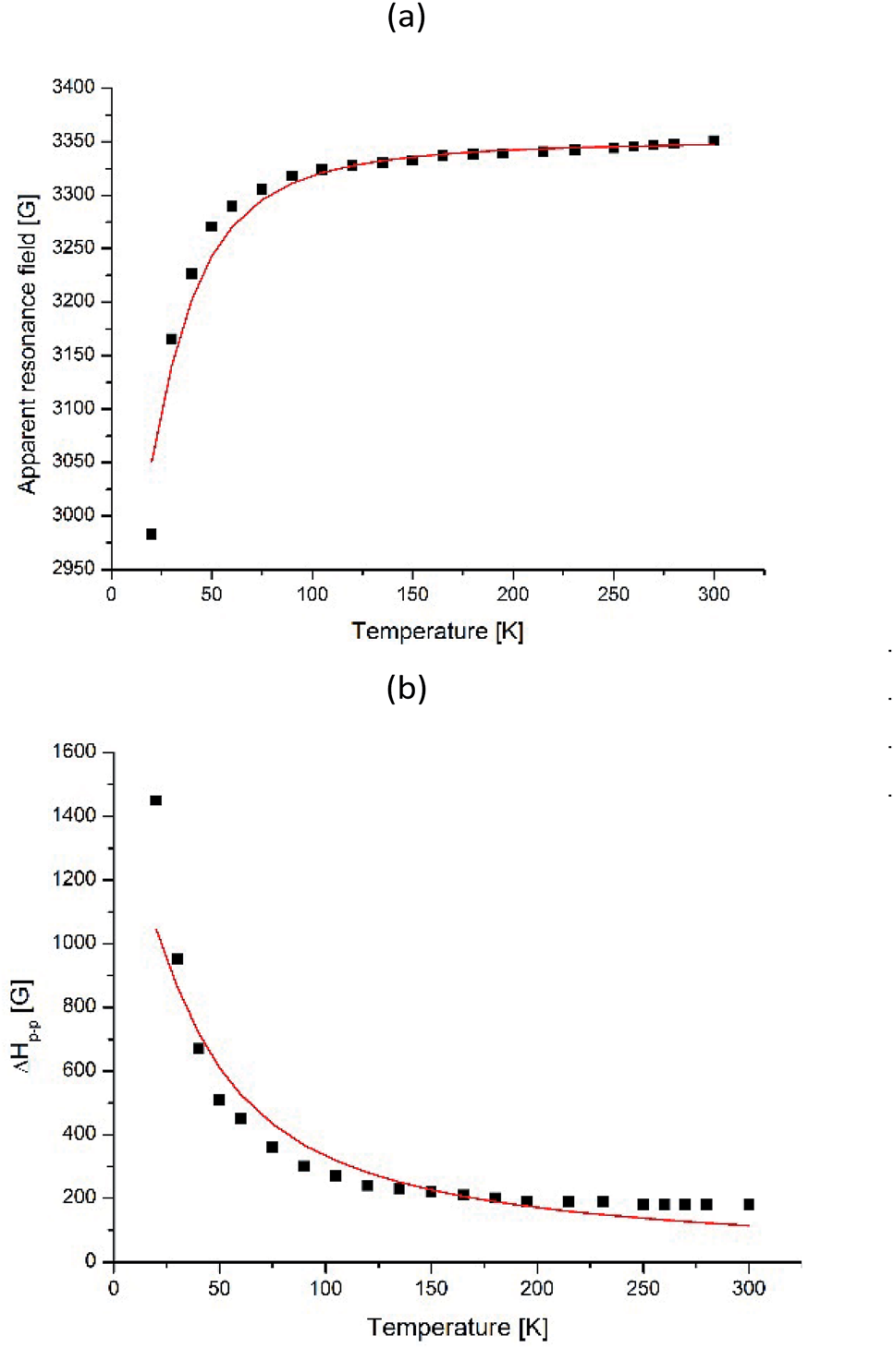
Temperature dependences of *H*_res_ (a) and Δ*H*_p–p_ (b) for I-type Fe(iii) centers for γ-Fe_2_O_3_ NPs in dendrimers. The solid line shows the theoretical dependence simulated by BK theory given by eqn (3) and (5) for (a) and (b), respectively.

The processes occurring in the system were analyzed according to the magnetic parameters obtained in the simulation. Knowing the ratio between the g-factor and the resonance field *H*_res_ = *hv*/*gβ*, *H*_res_(*T*) and Δ*H*_p–p_ for I-type centers are shown in Fig. 14. It can be seen that the resonance line broadens and shifts to the lower magnetic fields upon cooling. Such behavior is typical for superparamagnetic materials and is found in various magnetic NP systems.^45–51^

To understand the origin of the EPR line I-type in the powdered sample, we refer to the Raikher and Stepanov (RS) theory.^52^ The authors believed single-domain NPs to be magnetized uniformly and to possess a constant magnetic moment *μ* = *MV* (*V* – volume, *M* – magnetization of particle).

At low temperatures, when the anisotropy field is larger than the energy of thermal fluctuation of the magnetic moment (*k*_B_*T* < *KV*), the EPR line will be non-uniformly broadened in accordance with the random distribution of the effective anisotropic fields *H*_a_ (*H*_a_ = *2*|*K*|/*M*, where *k*_B_ – is the Boltzmann constant, *T* is the absolute temperature, and *K* is the effective anisotropy energy constant). On increasing the temperature, thermal fluctuations lead to a decrease of the effective anisotropy field, the temperature dependences of which are given by *H*_a_(*T*) = *h*_a_[1/*L*(*ξ*) − 3/*ξ*], in which *h*_a_ is the “true” anisotropy field, *ξ* = *MVH*/*k*_B_*T*, and *L*(*ξ*) is the Langevin function.

The observed EPR spectra provide an opportunity to estimate *h*_a_, *μ*, the mean diameter of NPs. *H*_res_ – is the value appropriate to the isotropic superparamagnetic at a high temperature. From the temperature dependence of the effective anisotropy field *H*_a_(*T*), which was determined to be *H*_res_–*H*_0_ we can observed the value of the anisotropy constant. As seen from equation *H*_a_(*T*) = *h*_a_[1/*L*(*ξ*) − 3/*ξ*], the character of the temperature dependence of the resonance field is connected in a straightforward way with the nature and sign of the NP magnetic anisotropy: as the temperature decreases, *H*_res_ increases for systems with *K* > 0 and decreases for systems with *K* < 0.

**Fig. 15:**
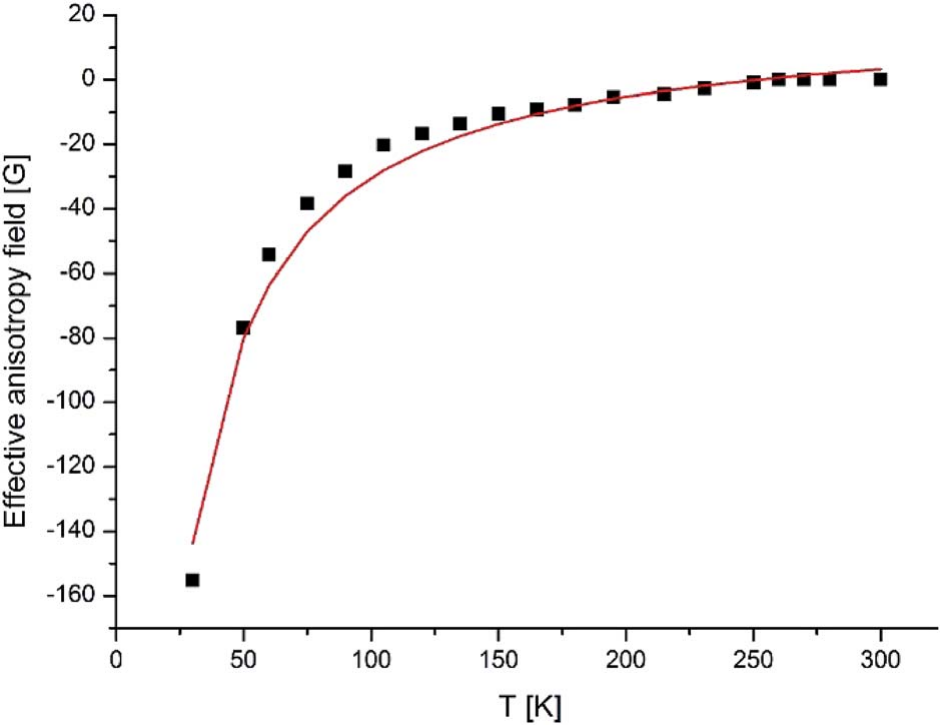
Temperature variation of *H*_a_(*T*) of γ-Fe_2_O_3_ NPs incorporated into dendrimers. The solid line shows the theoretical dependence simulated by RS theory.

The best fit of the experimental dependences is obtained with the following parameters, presented in the Table 6 for all four compounds and for the fourth generation dendrimer in Fig. 15.

**Table 6:** Calculated parameters using the Raikher and Stepanov theory^51^

Compound	*H*_0_, G	*MVH*/*k*_B_, K	*h*_a_, G	*μ*, *μ*_B_	*V*, nm^3^	*d*, nm
D1-K2.10-(Fe_2_O_3_)_1_	3317	90	−1284	402	9.59	2.63
D2-K2.10-(Fe_2_O_3_)_2_	3342	77	−1375	343	8.18	2.5
D3-K2.10-(Fe_2_O_3_)_5_	3346	45	−308	200	4.77	2.09
D4-K2.10-(Fe_2_O_3_)_10_	3351	30	−842	133	3.18	1.82

When *μ* is the magnetic moment of an individual nanoparticle and assuming a spherical form of the particle and taking *M* = 389 emu cm^−3^ for bulk γ-Fe_2_O_3_,^53^ we obtained values for the mean volume and mean diameter of the particles.

The data were analyzed using the Berger and Kliava (BK) approach.^54,55^ Berger *et al.* have also shown that the Landau–Lifshitz equation best describes the resonance spectra at different temperatures.^56^ Moreover, they took into account the demagnetizing field and the distribution of particles by diameters (volumes). Under typical EPR conditions (for the strong external magnetic field),^25^ the Zeeman energy predominates over the magnetic anisotropy and magnetostatic energies, so the effective field seen by an NP in the direction of the applied field (*H*_appl_) is simplified to eqn (2):

2 *H*_eff_ = *H*_appl_ + *H*_a_ +*H*_d_

where *H*_a_ is the anisotropy field and *H*_d_ is the demagnetizing field.

Berger *et al.*^55^ showed that *H*_res_ (corresponding to the absorption maximum) was given by eqn (3):3
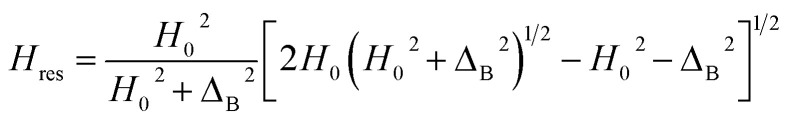


It can be seen, that when *Δ*_B_ increases – *H*_res_ decreases.^56^ On the other hand, the volume and temperature dependence of the individual nanoparticle line width^48^ can be described by eqn (4):

4 *Δ*_B_ = *Δ*_T_*L*(*MVH*_eff_/*k*_B_*T*)

where *Δ*_T_ is the saturation line width at temperature *T*, *L*(*x*) = coth *x* − 1/*x* is the Langevin function with *x* = *MVH*_eff_/*k*_B_*T* and *V* being the particle volume. To describe the rapid increase of the individual line width with the decrease in temperature, the BK theory also takes into account the thermal fluctuation-induced modulation of the magnetocrystalline anisotropy energy. This mechanism leads to the temperature dependence of *Δ*_T_. The resulting volume and temperature dependence of the individual line width is then given by eqn (5):5
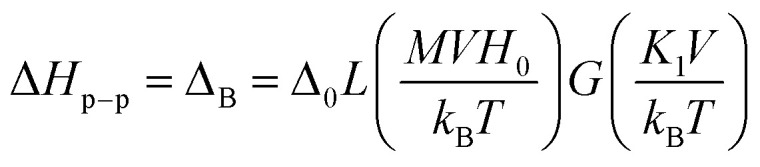
in which *Δ*_0_ is the saturation line width at 30 K for a particle of volume *V*_S_ (presumably the greatest volume in the statistical ensemble) and *G*(*y*_S_) is the function depending on the symmetry of the anisotropy field. For the case of axial symmetry, *G*(*y*_S_) is expressed by eqn (6):^48,57^6
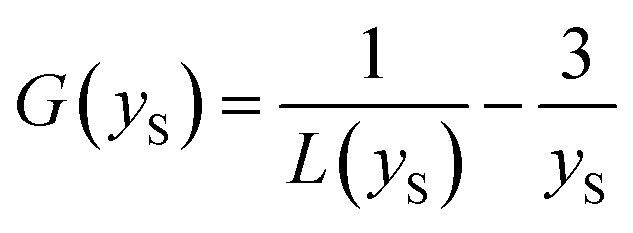
where *y*_S_ = *K*_1_*V*_S_/*k*_B_*T* is the ratio of the magnetocrystalline anisotropy energy to the thermal energy for a particle of volume *V*_S_ with the first-order anisotropy constant *K*_1_.

The results of modeling the temperature dependence of the resonance field and individual line width, using eqn (3) and (5) are illustrated in Fig. 15 for a fourth generation dendrimer. The theoretical dependencies have been calculated with the following parameters, presented in Table 7 for all the four compounds.

**Table 7:** Calculated parameters using the Berger and Kliava theory^54,55^

Compound	*H*_0_, G	*MVH*/*k*_B_, K	*K*_1_ *V*_S_/*k*_B_, K	*Δ*_0_, G	*μ*, *μ*_B_	*V*, nm^3^	*d*, nm
D1-K2.10-(Fe_2_O_3_)_1_	3337	165.4	9.1 × 10^3^	1900	742	17.6	3.23
D2-K2.10-(Fe_2_O_3_)_2_	3342	155.9	8.9 × 10^3^	1909	695	16.5	3.16
D3-K2.10-(Fe_2_O_3_)_5_	3346	126	7.2 × 10^3^	1680	564	13.4	2.95
D4-K2.10-(Fe_2_O_3_)_10_	3351	71	6.7 × 10^3^	1450	315	8.09	2.5

However, if we try to estimate the value of *V*_S_ from the second calculated parameter *K*_1_*V*_S_/*k*_B_, taking constant *K*_1_ equal to the magnetocrystalline anisotropy constant (*K*_V_ = −4.64 × 10^4^ erg cm^−3^) for bulk maghemite, we obtain an unreal magnitude of *V*_S_ = 1.997 × 10^4^ nm^3^ for the four generation dendrimer. It is clear that NPs fabricated inside dendrimeric matrices cannot have such a huge *V*_S_ volume, since the maximum particle sizes are limited by the cavity sizes of the dendrimer and iron oxide NPs formed in a dendrimer have a narrow size distribution.^58^ Therefore, we can assume that the NPs diameters have to be smaller.

Thus, for NPs encapsulated into a dendrimer we can assume that the anisotropy constant *K*_1_ cannot be equal to the magnetocrystalline anisotropy constant *K*_V_ of bulk maghemite, the *K*_1_ should be some orders larger than the magnetocrystalline constant. A significant increase in the anisotropy constant is commonly observed for NPs,^59–61^ and is attributed to the surface and shape contributions. Thus, both the RS and BK models showed a significant enhancement of the effective magnetic anisotropy constant dendrimeric (γ-Fe_2_O_3_) NPs as compared with bulk maghemite.

### Photophysical properties

The UV/Vis absorption and emission spectra were recorded in two solvents: dichloromethane and tetrahydrofurane. A quinine bisulfate solution in 0.1 N H_2_SO_4_ was used as a standard with known fluorescence quantum yield of 0.55 for the determination of dendrimers fluorescence quantum yield. The eqn (7) for calculating the fluorescence quantum yield:^62^

7 *φ*_x_ = *φ*_st_(*S*_x_/*S*_st_)(*A*_st_/*A*_x_)(*n*_x_/*n*_st_)^2^

where *φ*_x_ – the fluorescence quantum yield of the substance, *φ*_st_ – the fluorescence quantum yield of the standard (quinine bisulfate), *S* – the integrated fluorescence intensity (area under the spectrum), *A* – the absorbance at the excitation wavelength (239 nm for D1-K2.10-(Fe_2_O_3_)_1_, 254 nm for D2-K2.10-(Fe_2_O_3_)_2_, and 231 for D3-K2.10-(Fe_2_O_3_)_5_, D4-K2.10-(Fe_2_O_3_)_10_), *n* – the refractive index.

**Fig. 16:**
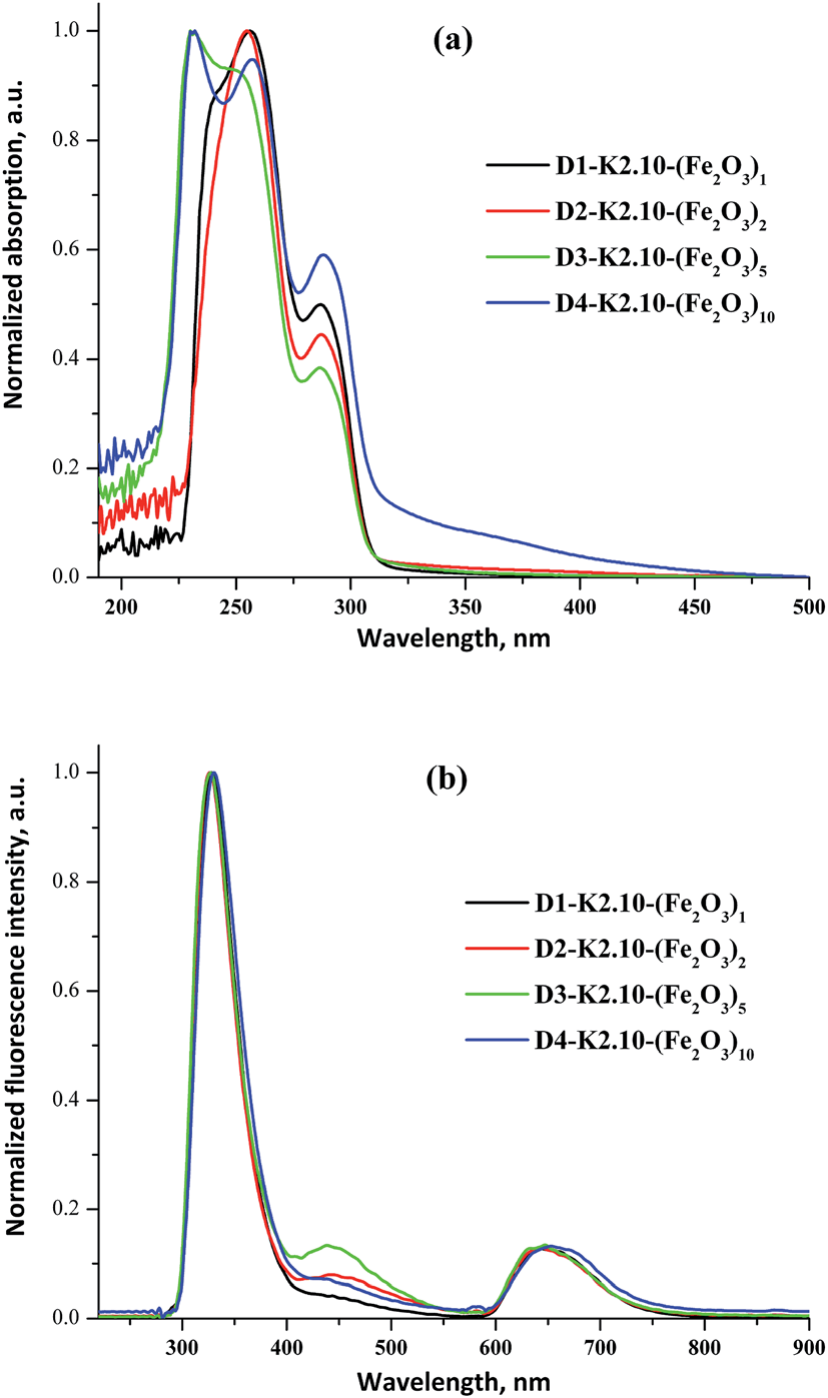
The normalized UV/Vis absorption (a) and fluorescence spectra (b) of D1-K2.10-(Fe_2_O_3_)_1_ in DCM and D2-K2.10-(Fe_2_O_3_)_2_–D4-K2.10-(Fe_2_O_3_)_10_ in THF (1 × 10^−6^ to 1 × 10^−5^ mol L^−1^).

The UV/Vis absorption and emission spectra of D1-K2.10-(Fe_2_O_3_)_1_ in DCM and D2-K2.10-(Fe_2_O_3_)_2_–D4-K2.10-(Fe_2_O_3_)_10_ in THF are shown in Fig. 16.

The optical data are summarized in Table 8. The absorption maxima of D2-K2.10-(Fe_2_O_3_)_2_–D4-K2.10-(Fe_2_O_3_)_10_ are significantly blue shifted compared to D1-K2.10-(Fe_2_O_3_)_1_, Fig. 16a.

**Table 8:** Photophysical properties of D1-K2.10-(Fe_2_O_3_)_1_–D4-K2.10-(Fe_2_O_3_)_10_^a^

Compound	*λ*_abs_, nm	*λ*_fl_, nm	*φ*, %
D1-K2.10-(Fe_2_O_3_)_1_	256	328	16
D2-K2.10-(Fe_2_O_3_)_2_	254	326	11
D3-K2.10-(Fe_2_O_3_)_5_	230	327	7
D4-K2.10-(Fe_2_O_3_)_10_	232	330	2

a *λ*_abs_, *λ*_fl_ – absorption and emission maxima, respectively.

An increase of the alkyl chain length (D1-K2.10-(Fe_2_O_3_)_1_ to D4-K2.10-(Fe_2_O_3_)_10_) leads to a decrease in the system polarization. This fact may be an explanation of the blue shift. The band at 248–257 nm corresponds to electronic transitions between the π–π* orbitals of the aromatic benzene rings. The band at 286–288 nm can be ascribed to n–π* transitions of the amide carbonyl group.^32^ The emission maxima of D1-K2.10-(Fe_2_O_3_)_1_–D3-K2.10-(Fe_2_O_3_)_5_ are at 328, 326, and 327 nm, respectively, which shows an identical variation trend to their absorption spectra, Fig. 16b.

The fluorescence quantum yields of D1-K2.10-(Fe_2_O_3_)_1_ in DCM and D2-K2.10-(Fe_2_O_3_)_2_–D4-K2.10-(Fe_2_O_3_)_10_ in THF are presented in Table 8. The D2-K2.10-(Fe_2_O_3_)_2_–D4-K2.10-(Fe_2_O_3_)_10_ fluorescence quantum yields are lower than D1-K2.10-(Fe_2_O_3_)_1_. These results should be attributed to an increase of the phenyl substituents number D1-K2.10-(Fe_2_O_3_)_1_ to D4-K2.10-(Fe_2_O_3_)_10_. The phenyl substituents rotation leads to a fluorescence quenching of the system because of an increase of non-radiative losses.

## Conclusions

Thus, stable DENPs derivatives of poly(propylene imine) from the first to the fourth generation with iron oxide were synthesized. These compounds are iron oxide nanoparticles encapsulated inside the dendritic matrix. All compounds obtained show mesomorphic properties forming a columnar hexagonal mesophase and vitrify at cooling.

The EPR spectra of the polycrystalline samples showed broadening and shifting of the EPR line to lower fields upon cooling. Such a behavior is typical for the superparamagnetic resonance of single-domain particles and was described by the RS theory. Quantitative analysis of the temperature dependence of the effective resonance field of the EPR line revealed an enhanced value of the effective magnetic anisotropy constant of NPs relative to bulk γ-Fe_2_O_3_, this was associated with the influence of surface and shape effects.

## Conflicts of interest

There are no conflicts to declare.

## Supplementary Material

RA-009-C9RA03732B-s001
