# Supplementary material for: Liquid crystalline poly(propylene imine) dendrimer-based iron oxide nanoparticles
Source: RSC Adv. 2019 Jul 22;9(39):22499–512. doi: 10.1039/c9ra03732b (PMC9066696; doi:10.1039/c9ra03732b)
Supplement: RA-009-C9RA03732B-s001 [file RA-009-C9RA03732B-s001.pdf]

Electronic Supplementary Material (ESI) for RSC Advances.
This journal is © The Royal Society of Chemistry 2019

Supporting Information

Liquid crystalline poly(propylene imine) dendrimer-based iron oxide nanoparticles

Gruzdev M.S., Chervonova U.V., Vorobeva V.E., Ksenofontov A.A., Kolker A.M.

Table SI 1

Conditions of DENPs synthesis

Compound	Reagents, mg (mmol)	Solvent, V, ml	T, ^oC	τ, h	Yield, %
D1-K2.10-$(Fe_2O_3)_y$	1-K2.10-$(FeCl_3)_{1.8}$, 200 (0.1) N_2H_2 96 (30) NaOH, 60 (15)	THF, 40 O_2	23	4.5	78.7
D2-K2.10-$(Fe_2O_3)_y$	2-K2.10-$(FeCl_3)_{4.7}$, 200 (0.049) N_2H_2 80.53 (27.53) NaOH, 55.8 (13.4)	THF, 50 O_2	23	4.5	58.3
D3-K2.10-$(Fe_2O_3)_y$	3-K2.10-$(FeCl_3)_{9.2}$, 400 (0.048) N_2H_2 72.53 (17.84) NaOH, 50.64 (11.8)	THF, 55 O_2	23	4.5	47.9
D4-K2.10-$(Fe_2O_3)_y$	4-K2.10-$(FeCl_3)_{21.1}$, 200 (1.02) N_2H_2 64.06 (20.02) NaOH, 40 (10.1)	THF, 57 O_2	23	4.5	38.7

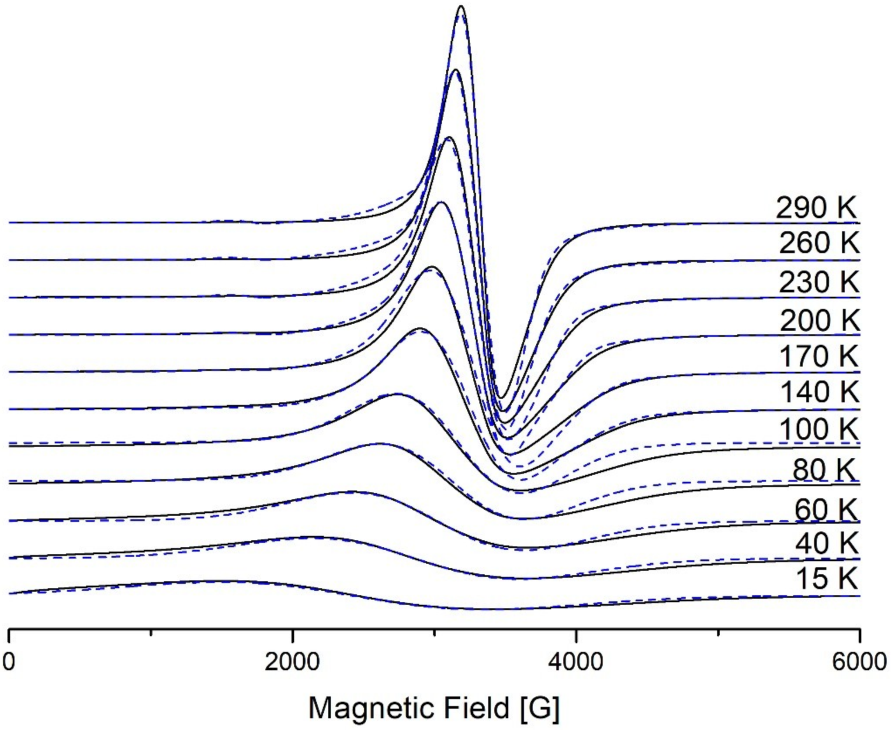

Figure 1 SI. Temperature dependence of EPR spectra for γ-Fe_2O_3 NPs encapsulated into the first generation dendrimer. All spectra were recorded under the same conditions. Dashed lines show the theoretical EPR spectra simulated by EasySpin Matlab.

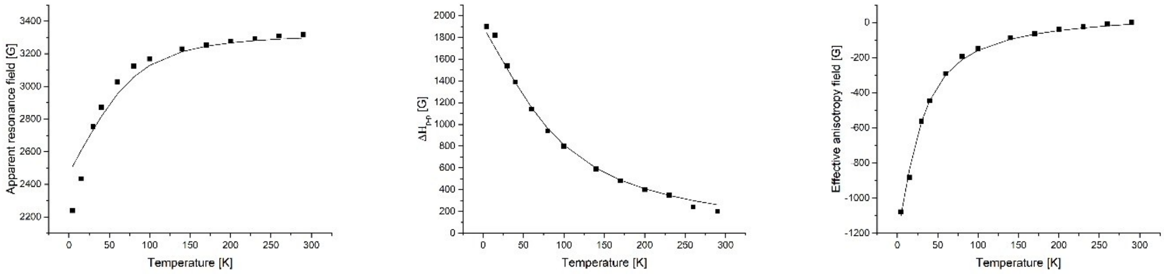

Figure 2 SI. Temperature dependences of H_{res} (a) and $\Delta H_{p\text{-}p}$ (b) and $H_a(T)$ (c) for I-type Fe(III) centers for γ-Fe_2O_3 NPs in the first generation dendrimer. The solid line (a,b) shows the theoretical dependence simulated by BK theory given by Equations (5) and (7) for (a) and (b), respectively. The solid line (c) shows the theoretical dependence simulated by RS theory.

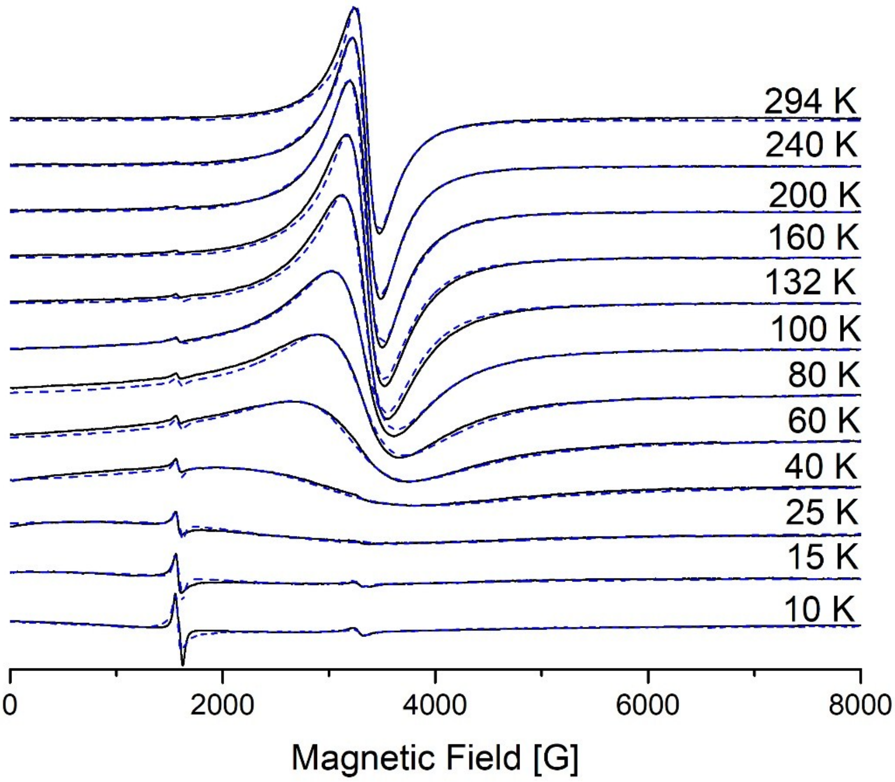

Figure 3 SI. Temperature dependence of EPR spectra for γ-Fe_2O_3 NPs encapsulated into the third generation dendrimer. All spectra were recorded under the same conditions. Dashed lines show the theoretical EPR spectra simulated by EasySpin Matlab.

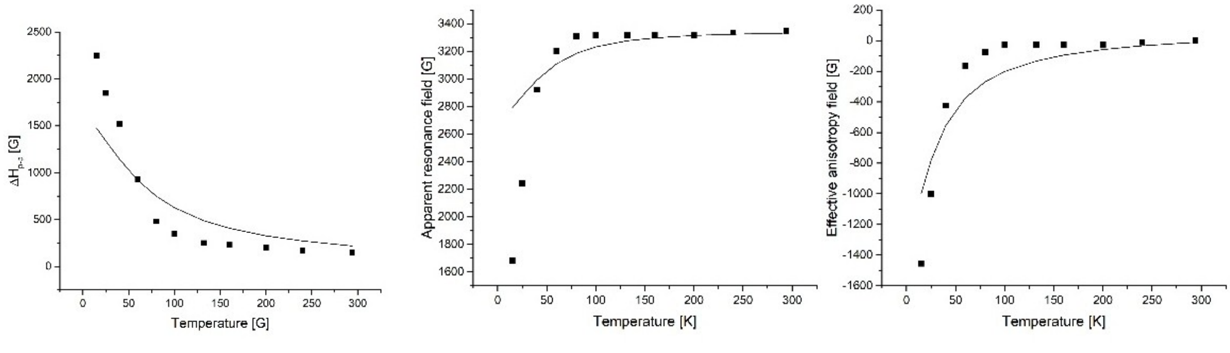

Figure 4 SI. Temperature dependences of H_{res} (a) and $\Delta H_{\mathrm{p\text{-}p}}$ (b) and $H_a(T)$ (c) for I-type Fe(III) centers for γ-Fe_2O_3 NPs in the third generation dendrimer. The solid line (a,b) shows the theoretical dependence simulated by BK theory given by Equations (5) and (7) for (a) and (b), respectively. The solid line (c) shows the theoretical dependence simulated by RS theory.
